# Atomistic Detail of the Formation of WSO and WSeO Janus Monolayers and Their Role for Cation Selection: Toward Effective Materials for Environmental Remediation

**DOI:** 10.1021/acsomega.5c03270

**Published:** 2025-09-20

**Authors:** Jonathan Guerrero-Sanchez, Dalia M. Muñoz-Pizza, Do Minh Hoat

**Affiliations:** † Centro de Nanociencias y Nanotecnología, Universidad Nacional Autónoma de México, km. 107, Apdo. Postal 14, Carretera Tijuana-Ensenada, C.P. 22800 Ensenada, Baja California, México; ‡ Facultad de Ciencias, Universidad Autónoma de Baja California, C.P. 22860 Ensenada, Baja California, Mexico; § Institute of Theoretical and Applied Research, 374802 Duy Tan University, Ha Noi 100000, Vietnam; ∥ School of Natural Sciences, 374802 Duy Tan University, Da Nang 550000, Vietnam

## Abstract

Transition metal dichalcogenides (TMDs), in their 2D form, are a family of semiconductors with great versatility and several applications, spanning from environmental, energy, electronics, and even spintronics. This work focuses on WS_2_ and WSe_2_ monolayers (MLs) and their oxidation process to obtain WSO and WSeO using quantum mechanical calculations. The oxidation occurs in well-ordered thermodynamically viable diagonal patterns. There is also a lattice parameter reduction with a linear behavior when going from WS_2_/WSe_2_ toward WSO/WSeO; this fact is directly related to the electronegativity of the chalcogen species (Se > S > O). All oxidized structures exhibited bonds with mixed covalent and ionic characteristics, with the W–O bond displaying the stronger ionic character. We tested the capacity of nonoxidized and oxidized monolayers as agents for ion trapping. Pristine WS_2_ and WSe_2_ monolayers effectively adsorbed Ca, K, Na, and Cl, with the lowest adsorption observed for Mg. In contrast, the Janus monolayers exhibited apparent selectivity toward cations and showed the lowest adsorption energy for the Cl anion under gas-phase conditions. Under aqueous conditions, the same behavior was observed for Ca, Mg, K, and Na, which form chemical bonds with the oxidized substrate while still coordinating with their hydration shells. In contrast, Cl interacts weakly with the surface, as it prefers to interact with its hydration shell. Electron localization function analysis demonstrated the ionic bond formation in the cations, and the noncovalent interaction index isosurfaces clarified the weak van der Waals (vdW) interactions that hold the Cl-surface interaction, being a strong proof that the oxidized part of the Janus WSO and WSeO monolayers evidence cation selection, which points to these 2D materials as potential anodes for water treatment.

## Graphical abstract

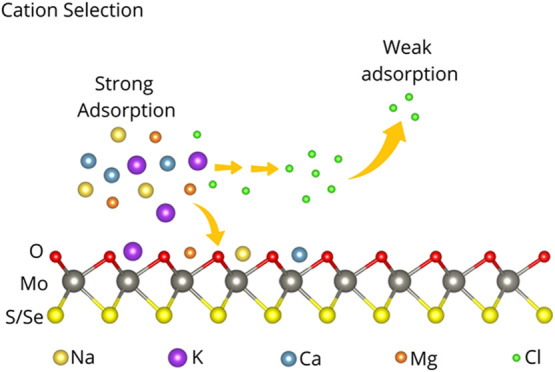

## 1 Introduction

The scientific community’s quest for ever-improving and smaller devices has led to the exploration of two-dimensional materials. These materials, with their large surface area and, in some cases, increased reactivity due to their natural dimensional confinement, present a promising avenue for nanotechnology applications, even in environmental remediation. Their versatility, demonstrated by their ability to be designed on the atomic scale to suit specific applications, opens up a wide range of possibilities. Common assessments, such as doping, stress, and shape alteration, are used to fine-tune the physical properties of these materials. There are several 2D material families; one stands out, the transition metal dichalcogenides (TMDs).

The TMD family includes several members. They are formed by sandwiching transition metals, such as Mo, W, Ti, Zr, and Hf, between two chalcogen layers (S, Se, or Te). TMDs are arranged in 1T, 2H, and 3R structures.^1^ The first has an octahedral arrangement, and the 2H and 3R have a trigonal prismatic arrangement. 2H and the metastable 1T are the most common in TMDs.^1^ Focusing on the 2H structure, 2D MoS_2_, MoSe_2_, WS_2_, and WSe_2_ emerge as the most important of the family. These materials exhibit direct semiconducting behavior, with band gaps in the visible range^2,3^ and enhanced photoluminescence.^4,5^ They have high surface area, high carrier mobilities, and high conductivity,^6^ are flexible and robust,^7,8^ and possess enhanced surface reactivity.^6^ These physical properties make them useful for several applications.

MoS_2_ has a diverse range of applications, including biomedical, energy storage, optoelectronic, sensors, catalysts, and environmental monitoring.^9^ MoSe_2_ is well-known for its applicability in the hydrogen evolution reaction, where it can be used in its pristine form, with defects, or with doping, or even in combination with other 2D materials.^10^ It can also be applied as part of supercapacitors and as an anode for lithium-ion or sodium-ion batteries.^11^ WS_2_ is also a versatile material, which finds numerous applications, including photocatalytic removal of contaminants or dyes, as a membrane for water treatment, toxic gas sensing, biomedical imaging, drug delivery, and several other uses.^12,13^ Finally, WSe_2_ is applied mainly in electronics, optoelectronics devices, and field-effect transistors.^14,15^

These materials are highly versatile but still open to improvement through the use of dopants, structural modifications, and even conversion to their Janus counterparts. The charge density asymmetry that emerges in the Janus counterparts generates a dipole due to the presence of species with different electronegativities on each side of the monolayer (ML), generating polarization and enhanced reactivity. This phenomenon can be applied in 2D ferroelectric materials and catalysis. The most famous Janus materials are MoSeS^16^ and WSeS.^17^ Still, it is possible to use Te or O for such modifications, as combining the different electronegativities in the materials brings enhanced properties through charge density asymmetry.^18^ In this sense, we recently assessed the formation and improvement of several Janus monolayers like MoSO, MoSeO, WSO, and WSeO.^19–22^ One step further in achieving Janus monolayers was recently accomplished by generating O-rich edges in MoS_2_, which enhances the selectivity for directly producing methanol from methane and O_2_ at room temperature.^23^ Very recently, we explained the step-by-step oxidation process to reach the Janus MoSO and MoSeO,^24^ in which we found that the oxidation follows a pattern, occurring by forming diagonal-like arrangements of oxygen atoms until the Janus monolayer (ML) is reached.

Additionally, incorporating oxygen induces a direct-to-indirect band gap transition at the early stages of oxidation, with the gap being reduced systematically.^24^ The importance of such studies relies on the improvement of reactivity mediated by the difference in electronegativities; for example, in MoSSe nanoflakes, the electronegativity difference generates improved curvature, which increases the reactivity of the Se side toward the hydrodesulfurization reaction.^18^ That is not it; the curvature is even more pronounced when incorporating the MoSO and MoSeO into the game, which generates not only reactivity at the borders but also at the center of the nanostructures.^25^ Considering this, it is essential to describe the oxidation processes in TMDs on an atomic scale. In this article, we focus on analyzing the formation of WSO and WSeO. The change in the structure and electronic properties in each oxidation step. We also analyzed the capacity of pristine and Janus monolayers to remove ions from seawater and brackish water. Our results demonstrate that the oxidized part of the Janus monolayers exhibits strong selectivity toward cations, forming chemical bonds with Ca, Mg, K, and Na in both the gas phase and under aqueous conditions. At the same time, Cl anions interact through weak van der Waals (vdW) forces with the oxidized part of the Janus monolayers both in the gas phase and under aqueous conditions, demonstrating that Janus WSO and WSeO may be efficient anode materials for water treatment.

## 2 Methods

The oxidation process in WS_2_ and WSe_2_ monolayers has been carried out using the Vienna Ab Initio Simulation Package.^26,27^ The electron–electron interaction is sampled using the generalized gradient approximation, with the parametrization proposed by Perdew, Burke, and Ernzerhof.^28^ The ion-electron interactions are treated using projector-augmented wave (PAW) potentials.^29^ Electronic states were expanded in plane waves with an optimized Kinetic energy cutoff of 450 eV. The structural relaxation was achieved when the energy differences reached 10^–4^ eV, and the force convergence was lower than 0.01 (eV/Å). The supercell method included a 15 Å vacuum to preclude self-monolayer interactions induced by the periodic boundary conditions. The irreducible Brillouin zone was sampled using 3 × 3 × 1 *k*-points for the 3 × 3 periodicities, while the electronic properties were obtained using 6 × 6 × 1 *k*-points. Spin–orbit coupling interactions are important in Janus TMDs when focusing on the emergence of spin–orbit-induced electronic effects, such as Rashba splitting;^30,31^ however, since our manuscript focuses on understanding cation selection in the gas phase and under aqueous conditions, we do not consider them. The primary reason is that we need to perform several optimizations and SOC is computationally expensive. Also, SOC is not expected to change the structural behavior observed in ion-surface interactions. For ion adsorption in the gas phase and under explicit solvent, we included van der Waals (vdW) interactions as modeled in the DFT-D3 Grimme approximation.^32^ For modeling ions under aqueous conditions, we added four water molecules surrounding monovalent Na and K, while six water molecules surround divalent Mg and Ca. In the case of the Cl anion, four water molecules surround it in the first hydration shell.

## 3 Results and Discussion

### 3.1 Stability and Structural Details under Oxygen Incorporation

This section analyzes the stability and structural evolution of the WS_2_ and WSe_2_ monolayers upon incorporation of oxygen in one side until reaching their Janus counterparts. These have already been reported.^20,22^ Still, the literature needs a step-by-step understanding of the oxidation process, so we incorporated oxygen atoms, one by one, in several sites and kept only the most stable. After calculations, we observed that oxidation occurs in well-defined diagonal patterns, as seen in Figure 1.

**Figure 1:**
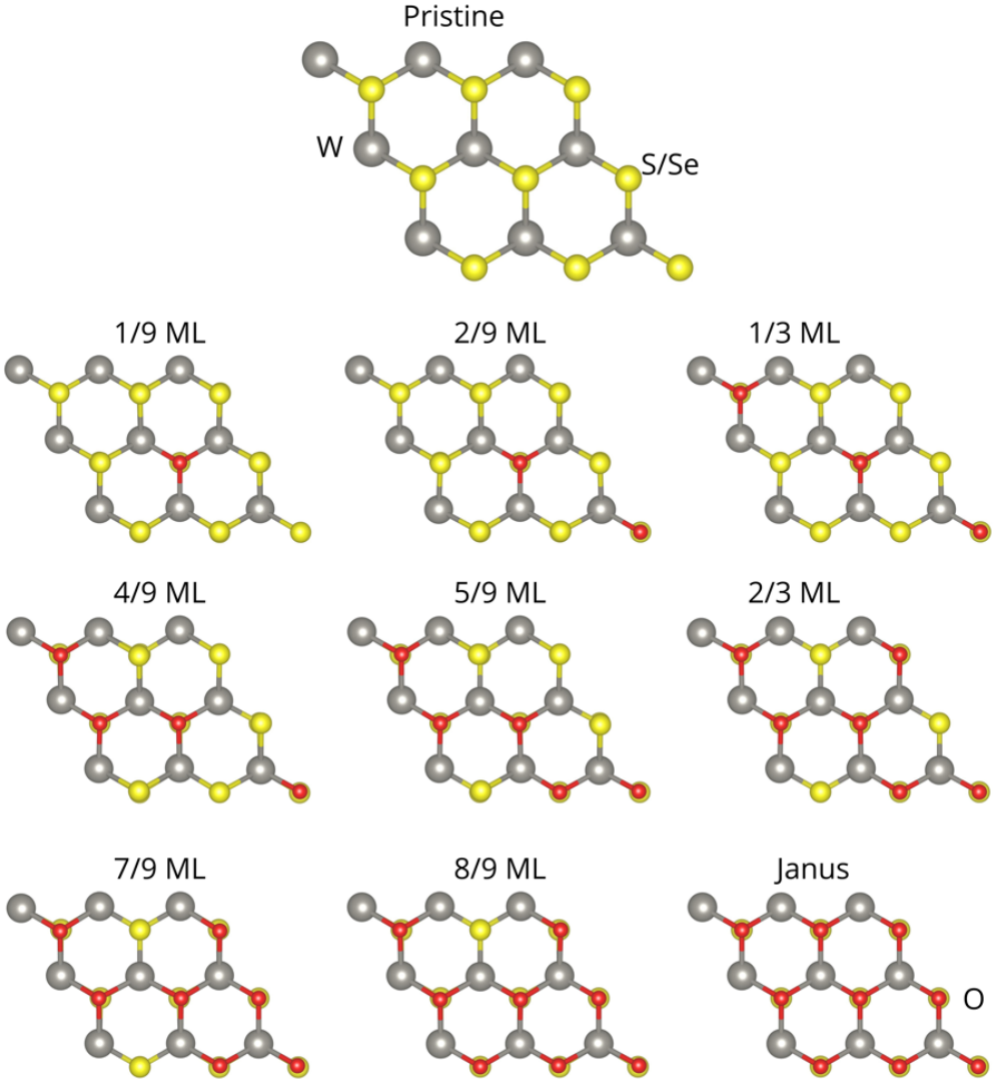
Pristine and all oxidation steps in WS_2_ (WSe_2_). Oxidation follows diagonal patterns. Gray spheres represent W atoms, yellow spheres represent sulfur or selenium atoms, and red spheres represent oxygen. ML stands for monolayer.

Since we are analyzing several coverages of oxygen in the monolayers, the total energy criterion is not suitable for determining stability; instead, the formation energy, which depends on the chemical potential of the incorporated species, is more suitable for analyzing the stability of oxygen incorporation. Zero is the reference in each case, which is either a WS_2_ or a WSe_2_ monolayer. The equation used to calculate the formation energy^33^ is

$$E_{f}=E_{system}-E_{ref}-n_{i}\mu_{i}-n_{O}\mu_{O}$$

where *E*_system_ stands for the energy of any WS_2_ or WSe_2_ monolayer systems with oxygen incorporation, *E*_ref_ is the reference energy that is either pristine WS_2_ or WSe_2_. *n*_O_ and μ_O_ define the number of oxygen atoms and their corresponding chemical potential, calculated as a molecule within a large cubic box of ∼20 Å. *n*_i_ and μ_i_ are the deficit of S or Se atoms in each case and their corresponding chemical potential, which was obtained considering the most stable bulk structure of each material. With that definition, any negative energy indicates a stable system. From Figure 2a, we can observe that even from the first oxygen incorporation (1/9 ML), the system is stable, and the stability increases as the oxygen coverage does. Also, oxygen incorporation is slightly more stable in WSe_2_; see red circles in Figure 2a.

**Figure 2:**
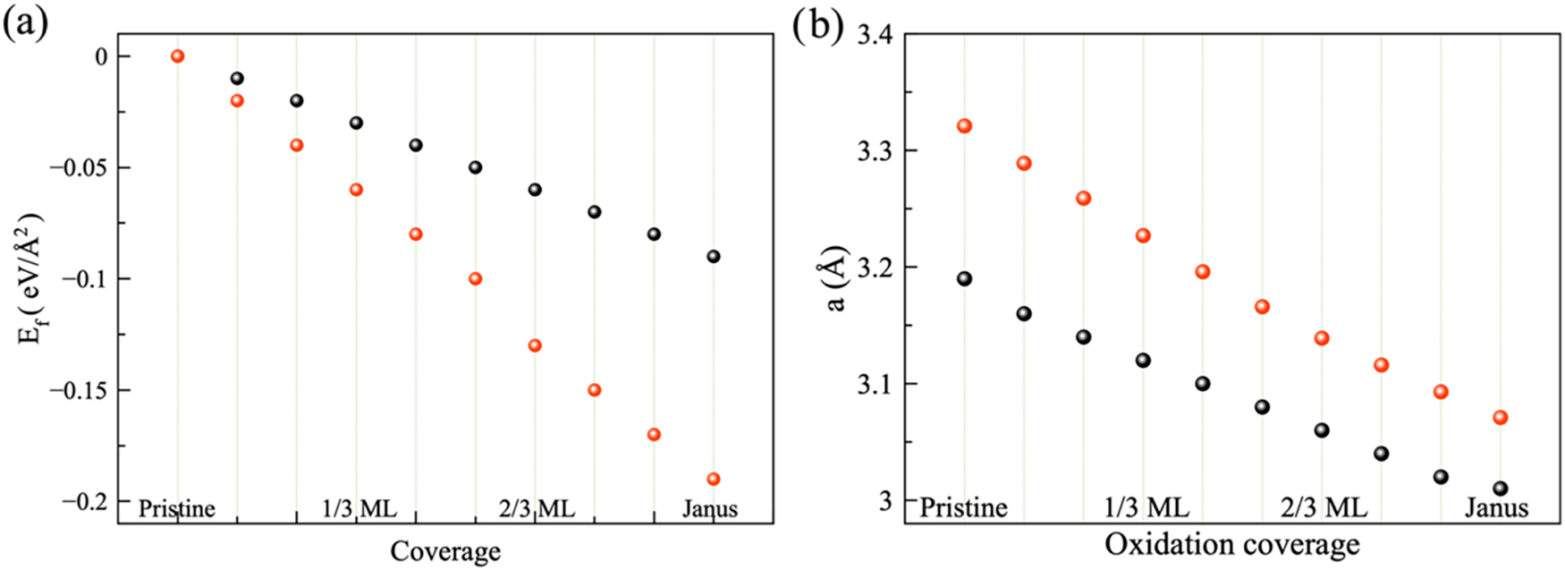
(a) Formation energies vs oxidation coverage and (b) lattice parameter evolution vs oxidation coverage. Black circles represent WS_2_ (WSe_2_).

Also, the monolayers were fully optimized in each oxygen incorporation without any constraints. Figure 2b depicts the lattice parameter evolution vs oxygen coverage. Black/red circles define the lattice parameter variation of WS_2_/WSe_2_. Notice that the lattice parameter gets reduced in each case; this is because the covalent radius of O (0.73 Å) is smaller than the one in S (1.02 Å)/Se (1.16 Å), so it is expected to have a large change in lattice parameter in the Se monolayer. For example, in the WS2 system, the lattice parameter changes from 3.19 Å in the pristine monolayer to 3.00 Å in the Janus WSO one, ∼6%. For the WSe_2_ monolayer, the lattice parameter changes from 3.32 Å in the pristine monolayer to 3.07 Å in the Janus WSeO, ∼7.5%.

### 3.2 Bonding and Electronic Properties

Once we described the structure evolution versus the oxygen content, we focused on the type of bonding formed by the interaction of O with W in each case. First, we focus on the WS_2_ with incorporated Oxygen. Different bonds are formed, O–W, S–W near the O, and S–W far from the O; see Figure S1, Supporting Information. Notice that the bonds exhibit a mixture of covalent and ionic characters, with the O–W bond being the one with the strongest ionicity. This is due to the large difference in electronegativity between O and W. Also, O affects the nearby S–W bond, which, due to the decompensation in W generated by O, possesses a slightly stronger ionicity than the S–W bond far from the O. So, as the difference in electronegativity is larger, the stronger the ionicity in the bond. A similar behavior is observed in WSe_2_ with incorporated oxygen, Figure S2, Supporting Information. Bonds are also a mixture of covalent and ionic. O–W bond possesses a stronger ionicity than Se–W bonds due to the large electronegativity of O.

Now, we discuss the effects of oxidation on the electronic properties of WS_2_ and WSe_2_. We are mainly interested in the band gap modification and transition from direct (WS_2_/WSe_2_) to indirect (WSO/WSeO). Figure 3 depicts the band gap vs oxygen coverage variation in both monolayers. Black (Red) circles in the figure depict the gap variation for WS_2_ (WSe_2_). In the case of WS_2_, the band gap decreases from 1.81 eV in WS_2_ to 1.57 eV in 2/3 ML, and then, it remains unchanged until the Janus monolayer is formed. In contrast, the case of WSe_2_ shows an increase in band gap for 1/9 ML from 1.55 eV in the pristine WSe_2_ to 1.61 eV and then to 1.69 in 2/9 ML. The observed difference in gap transition may be related to the fact that S and Se have different electronegativities and that O in the S matrix may abruptly change the electronic structure, while in the less electronegative Se atom, the local change in the electronic behavior is smother. Structural changes induced by lattice strain and charge redistribution as oxygen coverage increases modulate the band gap. After such coverage, it decreases almost linearly until 2/3 ML, which reaches 1.40 eV. Finally, from 2/3 ML to the Janus monolayer, the band gap experiences a slight reduction of about 0.05 eV.

**Figure 3:**
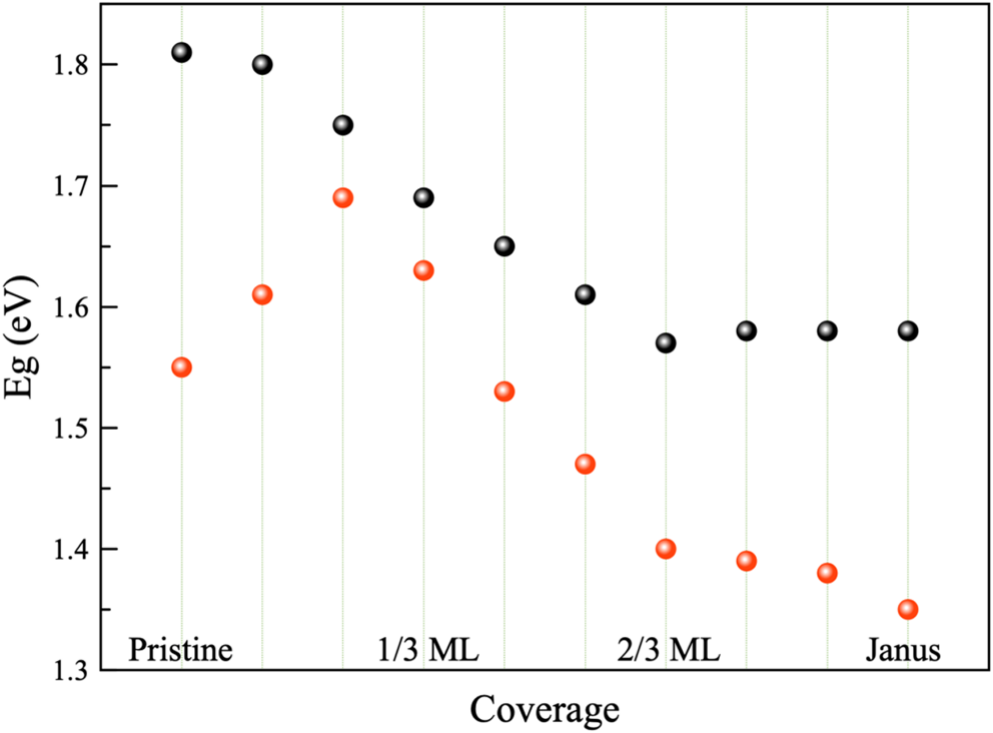
Band gap vs oxygen coverage for WS_2_ (black circles) and WSe_2_ (red circles).

We already analyzed the behavior of the gap vs Oxygen coverage in the monolayers; now, we focus on determining the band gap transition mediated by the oxidation degree, as depicted in Figure S3. Effective band structures were plotted in both cases using the band unfolding method proposed by Popescu and Zunger see^34^ and,^35^ which allows the unfolding of the band structure contributions of supercells of any size into the Brillouin zone of the primitive cell. In the case of WS_2_ and WSe_2_, unfolding was carried out from the 3 × 3 supercell to the 1 × 1 periodicity of the monolayer. We compared our unfolding results with those previously reported for WSO and WSeO in the 1 × 1 periodicity,^20,22^ and they agreed. Notice that for WS_2_, the band gap transition from direct-to-indirect occurs at a coverage of 1/9 ML (Figures S3 and 4a) and remains unchanged until the Janus WSO monolayer. Upon a thorough analysis of the gap versus oxidation effect on WSe_2_ (Figure S4), we observe that the transition occurs at 1/3 ML, not at 1/9 ML O (Figure 4b). After such coverage, the band gap remains indirect until the Janus WSeO monolayer.

**Figure 4:**
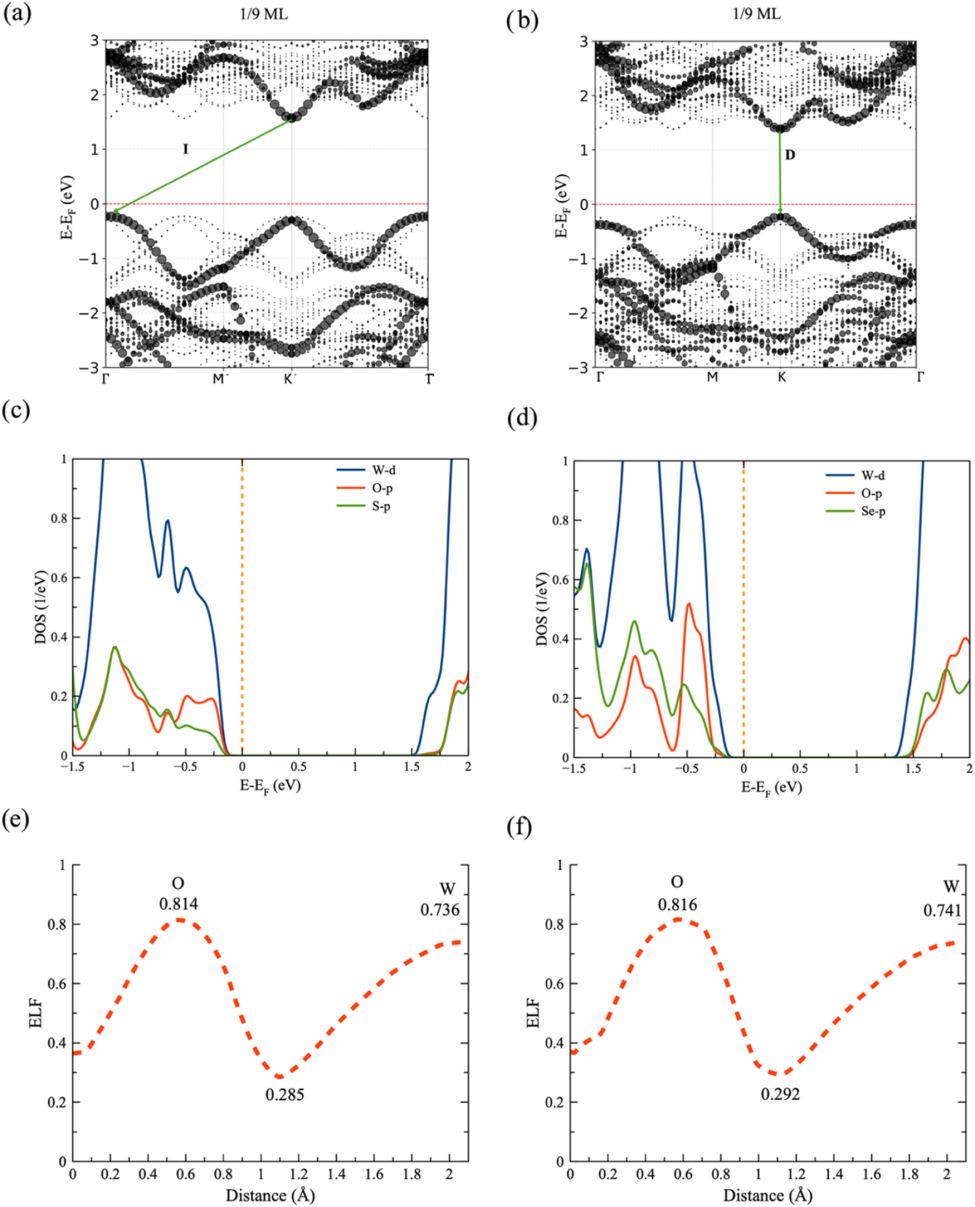
Effective band structure of (a) 1/9 ML O in WS_2_ and (b) 1/9 ML O in WSe_2_, (c) shows the projected density of states (PDOS) of the W-d, O-p, and S-p orbitals of the 1/9 ML O in WS_2_ model, and (d) W-d, O-p, and Se-p orbitals of the 1/9 ML O in WSe_2_ model. Electron localization function in the O–W bond is presented for each TMD in (e, f).

To investigate the distinct electronic behavior of WS_2_ and WSe_2_ with respect to oxygen incorporation, we analyzed the projected density of states (PDOS) for each oxidation step, focusing on the most significant contributions from W, S (or Se), and O atoms. The upper left panel of Figure S5 corresponds to the 1/9 ML O coverage in WS_2_. At this early oxidation stage. A noticeable hybridization around −0.5 eV is observed between the O, W, and S orbitals; this hybridization becomes more prominent in the 2/9 ML O model and persists until the full oxygen monolayer is reached in the Janus structure. In particular, oxygen states increasingly hybridize with both W and S orbitals in the energy range between 0 and −0.5 eV. As the oxygen reaches 4/9 ML of O, the O states begin to dominate the region near the Fermi level, overtaking the S contributions.

Figure S6 depicts the change in the electronic structure due to oxygen coverage in the WSe_2_ model. In the 1/9 ML O model, oxygen states do not significantly hybridize with the W orbitals near the Fermi energy; instead, they remain shifted toward more negative energies. This weak coupling continues in the 2/9 ML O case, where no strong orbital interaction is observed near the Fermi energy. A distinct change occurs in the 1/3 ML O coverage, where it is evident that the formation of an O–S–W hybridization occurs around −0.3 eV. This interaction is responsible for the observed transition from direct-to-indirect band gap. As coverage increases to 4/9 ML O, O orbital contributions begin to dominate more than Se states, and this remains until the complete O monolayer is achieved in the Janus structure.

To analyze the band gap transition in more detail at early oxidation stages in WS_2_, we examined the projected density of states of the O–W bond in conjunction with the electron localization function. We focus on the first oxidation step, 1/9 ML of the O in both systems, to elucidate the gap transition in WS_2_ and why it remains direct and increases in WSe_2_. In WS_2_, once we incorporate the first oxygen atom, the O-p states contribute significantly near the Fermi level (see Figure 4c), so there is a strong spectral overlap between O-p and W-d, which is a clear sign of strong hybridization. Such an interaction delocalizes the valence band maximum, effectively narrowing the band gap and inducing the gap transition.

In contrast, the first oxygen atom incorporated into the WSe_2_ monolayer generates electronic changes near the Fermi level, disrupting the Se-p and W-d hybridization. The O-p orbital contribution is relatively deep in energy (∼−0.3 eV), with almost no contribution near the Fermi level, as shown in Figure 4d. Therefore, the disrupted hybridization is not compensated by the O-p states, resulting in a more localized and energetically lowered valence band maximum, which in turn induces an increase in the band gap. The hybridization is compensated for when the coverage reaches 1/3 ML of O in WSe_2_, resulting in a band gap transition. The electron localization function analysis for both systems at 1/9 ML O coverage supports our assertions (Figure 4e,f), as it shows more localization in the W–O bond in WSe_2_ (ELF = 0.292) than in WS_2_ (ELF = 0.285). The slightly large covalency in the W–O bond for WSe_2_ with 1/9 ML of O coverage stabilizes the valence band maximum, shifting it to negative energies, which manifests as band gap opening due to hybridization suppression. While the slightly lower covalency in the W–O bond for WS_2_ with 1/9 ML of O coverage enables larger delocalization and band gap closing due to direct O-p contributions at the valence edge.

Once we know that the oxidized and Janus monolayers possess a combination of covalent and ionic bonds and that they are indirect band gap semiconductors at almost all oxygen coverages, we describe the effect of oxygen incorporation on the reactivity of the WS_2_ and WSe_2_ monolayers by using electrostatic potential isosurfaces (EPI). EPIs indicate charge density accumulation (red) or depletion (blue); in this regard, the system may be able to interact with other materials or molecules of different EPIs. For example, zones with charge density accumulation in the monolayer may interact with the zones of positive electrostatic potential in distinct molecules.

Figure 5a depicts the EPIs for WS_2_, 1/3 ML of O, 2/3 ML of O, and the Janus WSO monolayer. Due to O’s large electronegativity, there is a large charge density accumulation in the sites where O replaces S, while W sites remain almost neutral. For example, in the 1/3 ML O system, the diagonal formed by the oxygen incorporations possesses a larger reactivity than the rest of the monolayer; the same behavior is observed in 2/3 ML O. Finally, in WSO, there is a large charge density asymmetry, with one side of the monolayer formed by S (orange isosurface) and one by O (red isosurface), which confers the system a high reactivity in the O side. In the case of WSe_2_, Figure 5b, the Se atoms seem to have a lower charge density accumulation, which is now more evident in W. This is related to the similar electronegativity between W and Se. The scenario changes when incorporating O; for example, see the 1/3 ML O system, in which by the O incorporation, the charge density accumulation is now in the O sites, while the Se sites remain as in the pristine structure. The charge density asymmetry strengthens as the O coverage increases to 2/3 ML and reaches its maximum at the Janus WSeO monolayer (Figure 5b). The high charge density accumulation on the O side of the monolayers can be used to trap molecules or ions; therefore, it can be used in air and water remediation applications.

**Figure 5:**
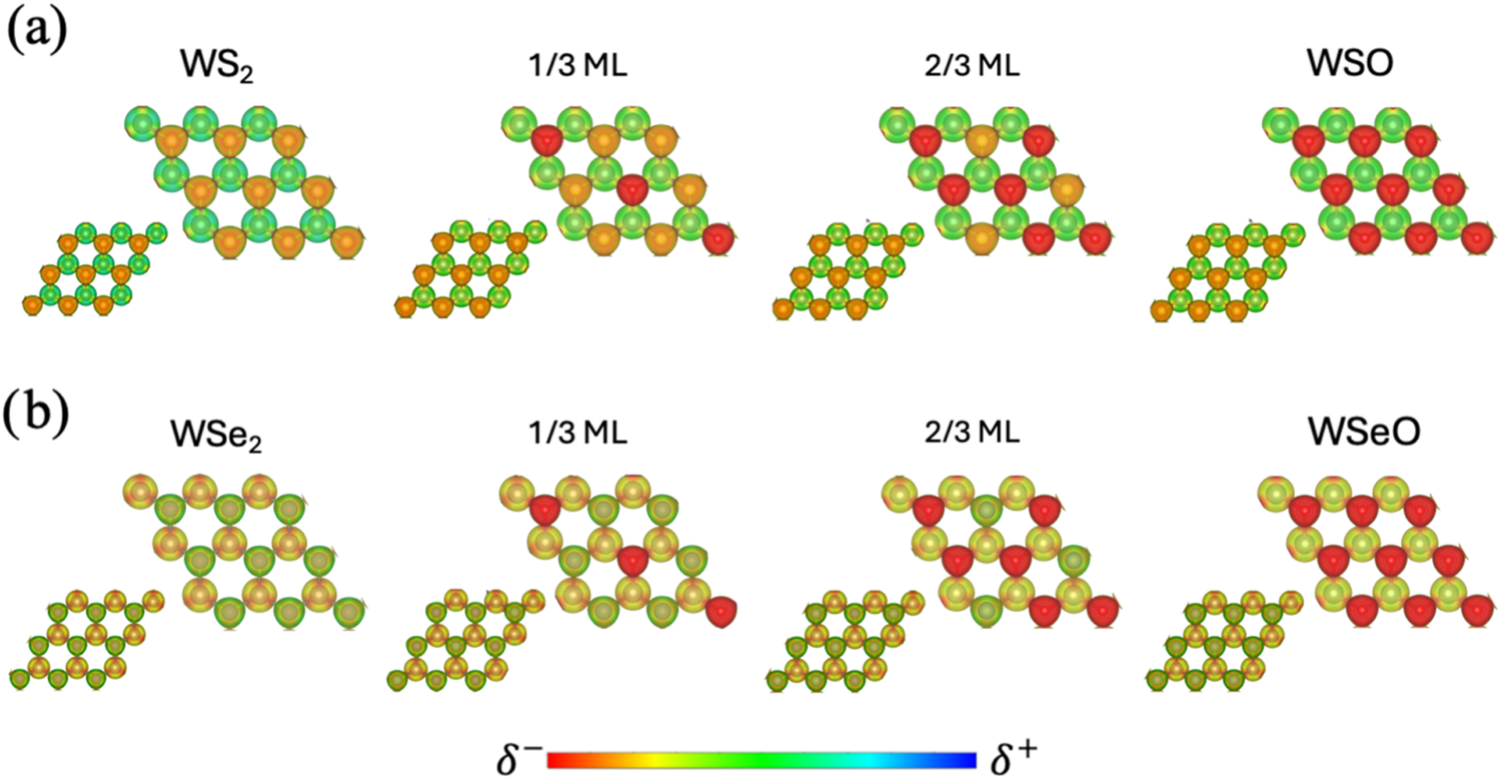
Electrostatic potential isosurfaces for different oxygen coverages in (a) WS_2_ and (b) WSe_2_. Red represents charge accumulation (negative potential), green represents neutral potential, and blue represents charge depletion (positive potential). The inset in the lower left part of each model shows the monolayer site without any oxygen incorporation.

### 3.3 Ion Adsorption on the Pristine and Oxidized Monolayers

Water remediation in a sustainable manner is one of the major challenges, as treatments are characterized by the use of highly expensive materials, complex maintenance, and high operational energy costs.^36^ Additionally, components such as membranes and filters have a relatively short lifecycle. In nanotechnology, the key aspect is removing either cations or anions using the same material, and this material must be stable enough to withstand prolonged use in nanofiltration. 2D materials are gaining interest due to their large surface area and capability to trap large quantities of ions for capacitive deionization (CDI).^37^ CDI is used for water desalination. It is an emerging technology that removes ions from brackish or seawater.^38^ Two electrically charged electrodes form the device; water flows between them, allowing the charged electrodes to separate cations or anions from the flow.^39^ CDI devices may also feature ion exchange membranes, which permit the flow of either cations (cation exchange) or anions (anion exchange).^40^ The 2D materials must be well-dispersed in the electrodes to achieve their maximum effectiveness. Typically, carbon nanotubes are employed to prevent restacking in monolayers,^41,42^ thereby maximizing the active surface area for ion trapping.

In this section, we use WS_2_, WSe_2_, WSO, and WSeO monolayers as vehicles to trap different ions present in seawater and brackish water. All adsorption models are presented in Figure 6a,b, as well as Figures S7–S10. The key point is to study Janus monolayers in desalination, specifically the removal of ions from brackish water or seawater. To do that, we adsorbed several ions (Na, K, Ca, Mg, and Cl) that usually have different oxidation states when dissolved in water. In this case, we treated it as an atomic species that adsorbs onto the monolayer. Figure 7 presents a heatmap comparison of the adsorption models considered for the WS2 (upper left), WSO (upper right), WSe2 (lower left), and WSeO (lower right) monolayers. Color indicates adsorption energy strength, where red is the lowest adsorption energy and blue is the maximum energy value found, that is, Ca adsorption on a WSeO monolayer H3 site (*E*_ads_ = −2.21 eV). Notice that the pristine monolayers exhibit more moderate adsorption energies, with values ranging from −0.23 to −1.17 eV, which depend on the ion and site. Also, notice that Cl ions have adsorption energies almost twice those of the Mg ions. In terms of stability, for WS_2_, the most stable adsorption site is T4 for Na, K, Ca, and Mg (Figure 6c). Although Cl stabilizes at the T4 site, the adsorption energies in the H3 and Top sites are very similar, 0.02 eV on the T4 site and 0.04 eV on the Top site for the WSO monolayer, while the energy difference for Cl on the T4 site is 0.01 and 0.02 eV when adsorbed on the Top site for WSeO. Such similar adsorption energies in Cl evidence the anion’s capability to diffuse through the Janus monolayer surface, contrary to the case in the pristine monolayers, where the adsorption energy is larger.

**Figure 6:**
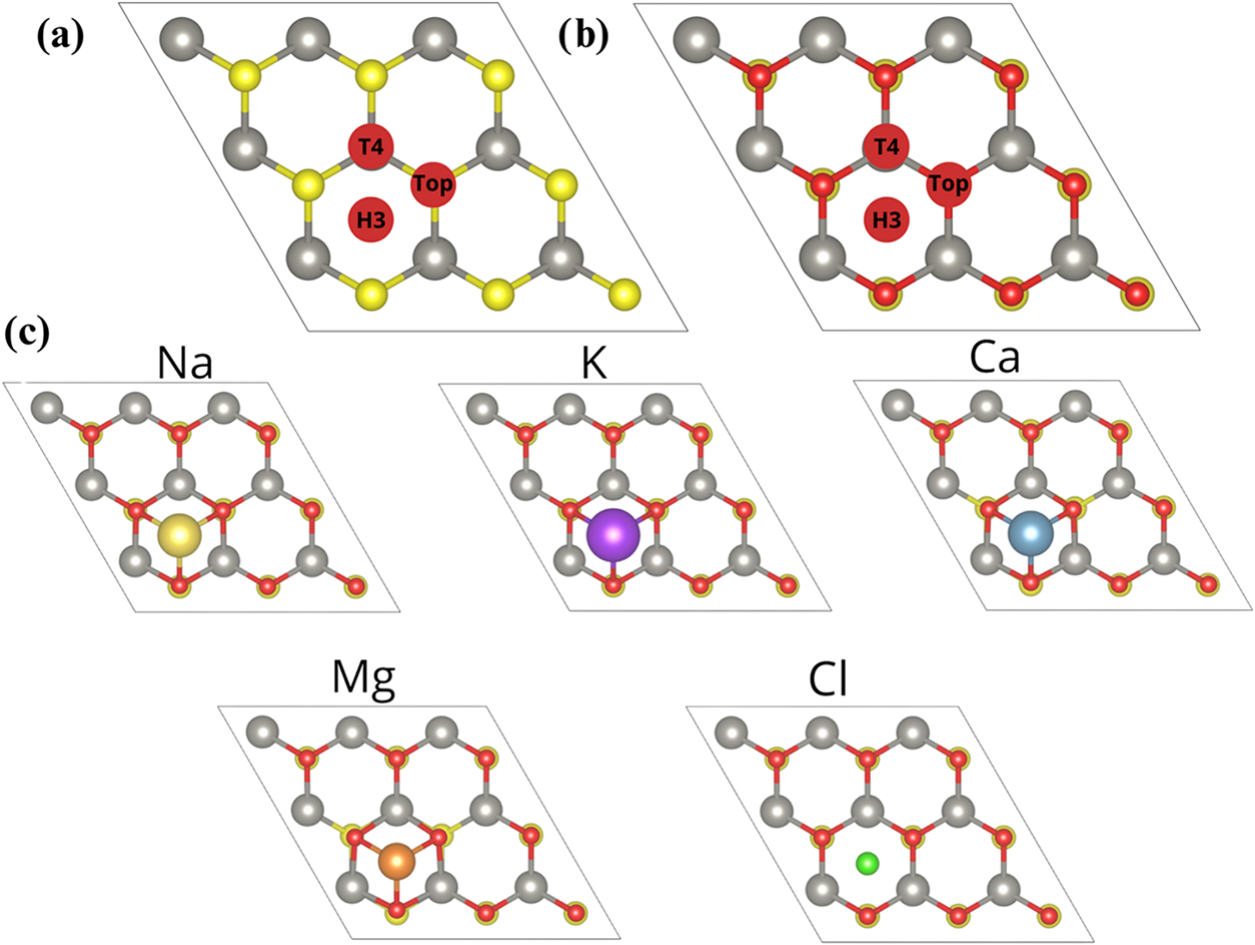
(a) High symmetry sites for the ion adsorption in the WS2 and WSe2 monolayers, (b) high symmetry sites for the ion adsorption in the WSO and WSeO monolayers, and (c) most stable model for each ion adsorption. Gray, red, and yellow stand for the W, O, and S/Se atoms. Ions are labeled in each subfigure of (c).

**Figure 7:**
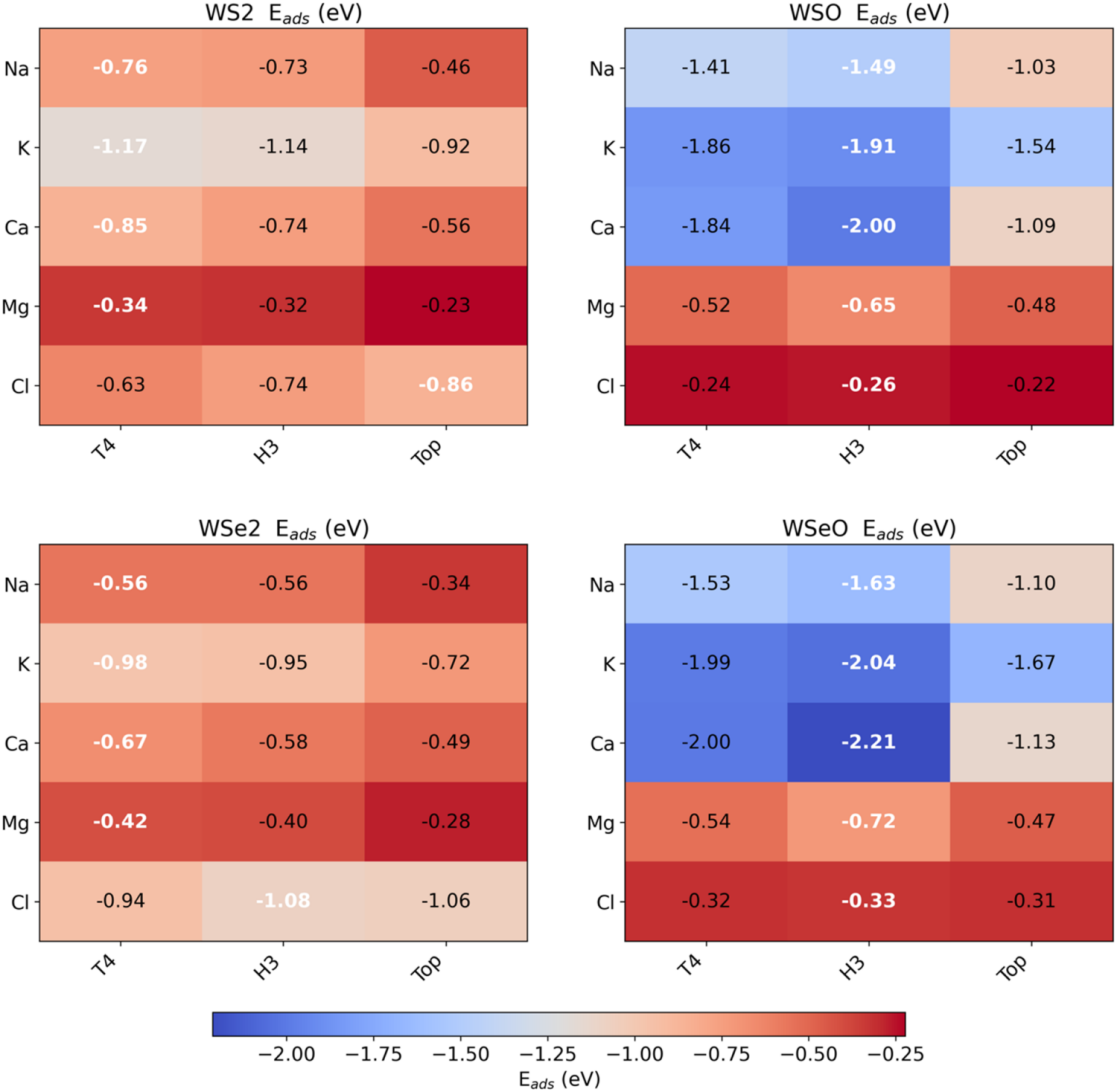
Heatmap of comparing the adsorption energy of all models treated in the pristine (MoS_2_ and MoSe_2_) and Janus (MoSO and MoSeO) monolayers.

Another important finding that can be observed from the data is that the adsorption strength is improved on the Oxygen side of the Janus monolayers. Cations are effectively trapped by all monolayers. But a difference emerges when treating Cl. Either WS_2_ or WSe_2_ present large adsorption energies (Figure 7); in contrast, the adsorption energies for Cl on the Janus monolayers (WSO and WSeO) are the lowest of all series, which evidence a clear cation selection mediated by the oxidized layer. This is not observed in the pristine counterparts, which opens the possibility of considering Janus monolayers as possible electrodes for cation removal in desalination devices. Additionally, due to the large electronegativity of the Oxygen layer, it is expected that H_2_O molecules interact primarily through weak van der Waals interactions with the Janus monolayers. Consequently, the possibility that Cl and H_2_O poison active sites used by the cations is low; see Figure 7. The higher activity of the Janus monolayers toward ions agrees well with the previously obtained electrostatic potential, which demonstrated that the oxygen side of the Janus monolayers possesses a high electron accumulation, followed by a large disposition to receive electrons from electropositive species like Na, K, Ca, and Mg. Additionally, the adsorption energies of Cl on the Janus monolayers are very small because Cl is highly electronegative and not prone to sharing electrons with monolayers that exhibit high electron accumulation; see the right part of Figure 5a,b. Studies are still needed to evaluate the toxicity and costs associated with using these materials in nanofiltration technologies.

### 3.4 Ion Trapping under Aqueous Conditions

Various monovalent or divalent salts, such as NaCl, KCl, MgSO_4_, MgCl_2_, CaCO_3_, and Na_2_SO_4_, are present in seawater and brackish water, which are targets for desalination.^43^ However, they do not remain as compounds but as cations and anions surrounded by hydration shells. Typically, monovalent cations, such as Na and K, are surrounded by four water molecules in their first hydration shell, forming a tetrahedral structure.^44^ In some cases, the hydration shell can comprise up to six water molecules. For example, six water molecules form a well-defined octahedral structure as the first hydration shell around Mg. The larger hydration shell is present in Ca, the largest cation, and can host from eight to ten water molecules.^44^

As a proof-of-concept under more realistic conditions, we compare the adsorption of the Cl anion and the cation with the lowest adsorption energy, Mg (Figure 7), under aqueous conditions in the WSO and WSeO monolayers (Figure 8). The adsorption of the remaining ions is presented in the Supporting Information (Figure S11). Figure 8a shows the Cl adsorption when solvated in water on WSO; notice that the interaction with the oxidized monolayer is weak, with a Cl–O surface distance of around d_Cl–O(s)_ = 3.6 Å, which is 0.35 Å larger than that when the Cl atom is adsorbed on the oxidized Janus monolayers. So, Cl prefers to remain solvated (in tetrahedral coordination) under aqueous conditions and interacts weakly with the monolayer. On the other hand, in the case of Mg, notice that it prefers to adsorb with the monolayer in a single bond on top of the surface oxygen atom. This result is expected since Mg retains an octahedral coordination structure similar to the solvated one. In this case, Mg coordinates with five water molecules and one surface atom, as shown in Figure 8b. The bond distances between Mg and the water molecules range from 2.11 to 2.16 Å, while the Mg–O surface distance is d_Mg–O(s)_ = 2.01 Å, demonstrating a stronger interaction with the Janus substrate. Upon comparing the Mg surface distances when just adsorbing the Mg atom (2.11 and 2.13 Å) with the ones under aqueous conditions (2.01 Å), we notice that under aqueous conditions, the Mg–O­(surface) bond is shorter by 0.11 Å, which indicates a stronger bond. Although Mg forms a bond with the surface under aqueous conditions, it is also noteworthy that its surrounding water molecules stabilize the Mg ion through weak van der Waals interactions with the substrate. So, the Janus WSO and WSeO monolayers in the oxidized part are expected to be potential anodes for cation selection. Similar results were found for the remaining cations (Na, Ca, and K), see Figure S9a–c in the Supporting Information.

**Figure 8:**
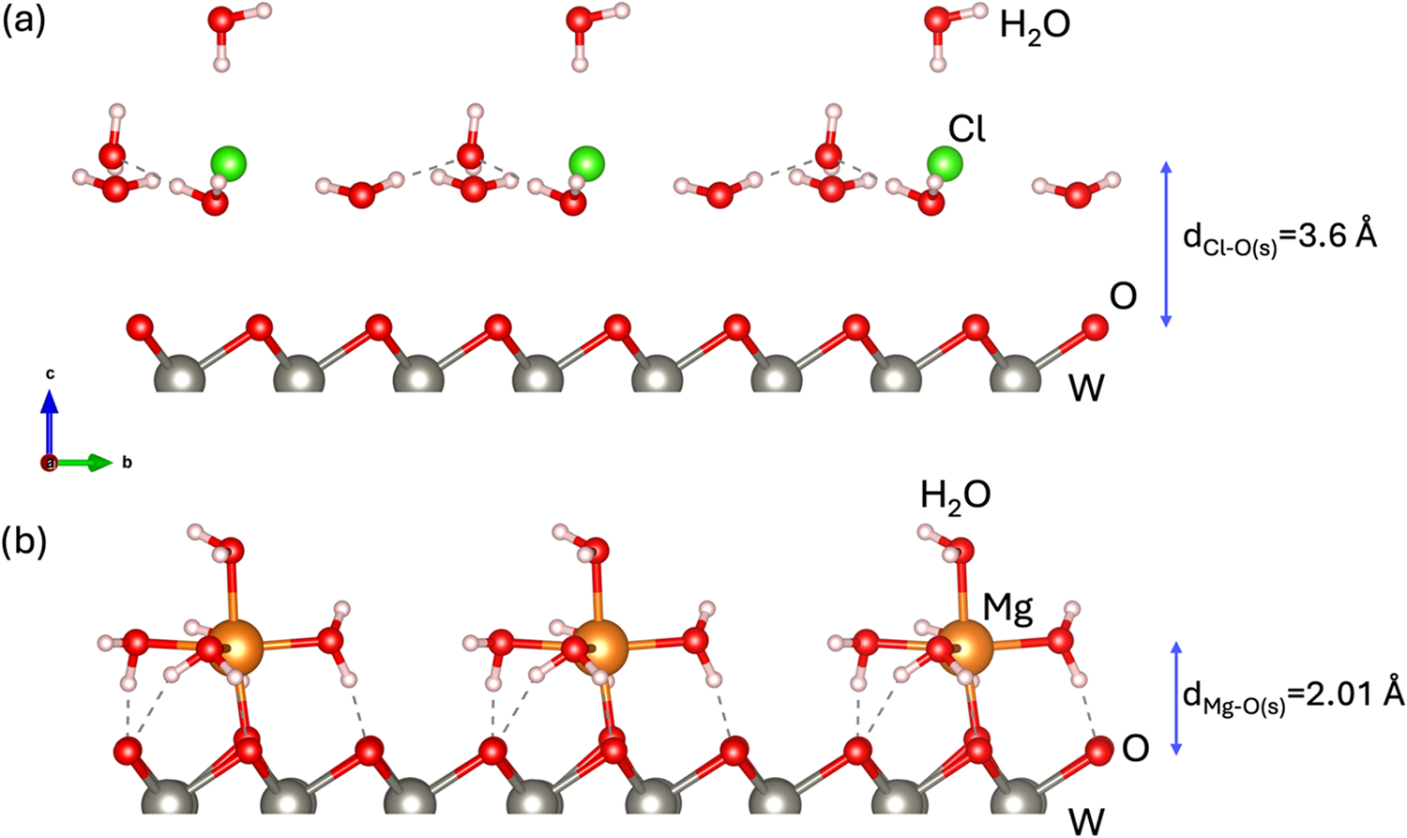
(a) Cl adsorption and (b) Mg adsorption under aqueous conditions. Gray spheres represent W atoms, yellow spheres represent sulfur or selenium atoms, and the red ones are oxygen.

### 3.5 Ion Bonding under Aqueous Conditions

In this subsection, we plotted the Electron Localization Function profiles for the cations interacting with the Janus substrate and the hydration shell. Focusing on the 2+ cations (Ca and Mg), we can observe that they exhibit different coordination behaviors. Ca coordinates seven bonds, forming three bonds with the surface and four bonds with the hydration shell. The O surface-Ca bond ELF profile shown in Figure 9a evidences a strong ionic behavior due to electrostatic interactions. In this configuration, Ca donates 1.63 e. In the case of Mg, which forms an octahedral coordination (one bond with the surface and five with the hydration shell; Figure 9b), we can observe a strong electrostatic interaction, localized mainly at the O surface atom. In this case, Mg donates 1.69 e between the surface bond and the hydration shell. Notice that both cations remain as 2+ species. Focusing our attention on 1+ cations, we can see that K forms a slightly distorted tetrahedral coordination shell (Figure 9c), with one surface O–K bond and three coordination bonds with the hydration shell. In this case, the surface O–K bond is still strongly ionic but less polarized. In this case, K donates 0.90 e to the system, confirming its cationic behavior. Finally, the Na cation forms a less distorted tetrahedral coordination with one surface O atom and three water molecules. From Figure 9d, we can see an ionic bond between Na and the O surface atom; this is confirmed by the electron donation present in Na, which is 0.89 e.

**Figure 9:**
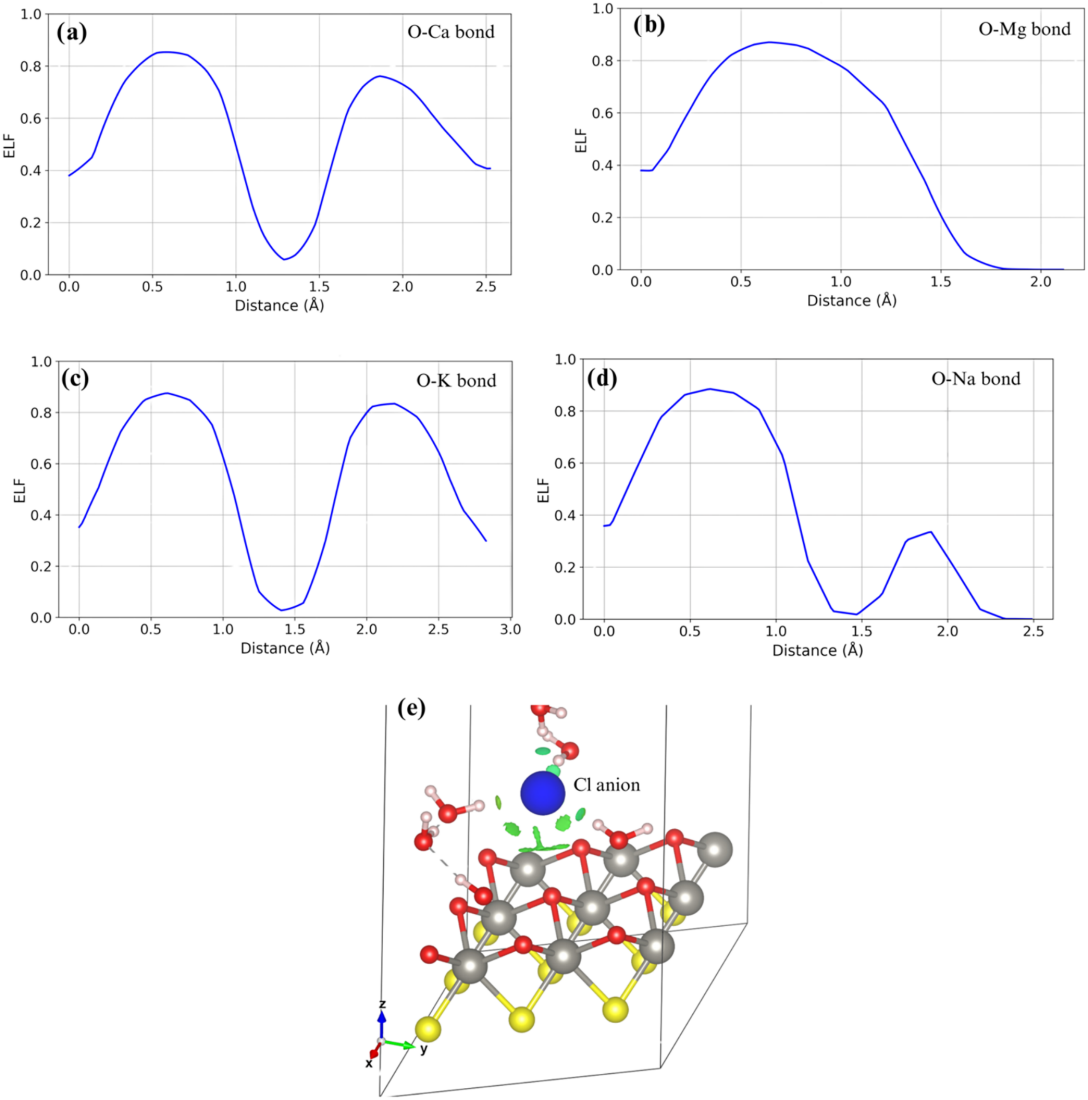
Electron localization function of (a) a surface O–Ca cation bond under explicit solvent, (b) surface O–K cation bond under explicit solvent, (c) surface O–Mg cation bond under explicit solvent, (d) surface O–Na cation bond under explicit solvent, and (e) noncovalent interactions index showing the isosurfaces of the weak interactions between surface O atoms and Cl anion. In panel (e), the blue circle represents the Cl anion, gray for W, yellow for S or Se, and red for oxygen atoms.

To analyze the Cl-surface interaction, we determined the noncovalent interactions index following.^45,46^ This index helps us understand the kind of weak interaction that appears between Cl, the hydration shell, and the substrate. Red stands for nonbonding or steric effects, green stands for weak van der Waals interactions, and blue accounts for bonding interactions. Figure 9e shows the noncovalent interaction index of the interaction between Cl and its surroundings. In sharp contrast to the cations, Cl anions form weak van der Waals bonds to the surface and remain with tetrahedral coordination with the water molecules. In this case, the Cl atoms donate 0.58 e to the coordination shell, and there is no electron donation to the substrate, as evident in Figure 9e, which depicts the noncovalent interactions index with isosurfaces related directly to van der Waals interactions. Notice also that hydrogen bonds exist between Cl and the water coordination shell.

The previous analysis allows us to prove that, effectively, the Janus monolayers exhibit a clear selectivity toward cations, in gas phase and under aqueous conditions.

## 4 Conclusions

In this article, we report on the oxidation process of WS_2_ and WSe_2_ monolayers to obtain their Janus counterparts, WSO and WSeO, using ab initio calculations. A proof-of-concept study was conducted to assess the ability of Janus monolayers to trap cations present in brackish and seawater selectively. First, we calculated the WS_2_ and WSe_2_ monolayer structures and properties to further oxidize one side of the monolayer to reach WSO and WSeO, respectively. Formation energies evidenced that each oxidation step is stable; also, since the covalent radius of O is smaller than that of S/Se, the lattice parameter in the Janus structures contracted by ∼6% in WSO and ∼7.5 in WSeO, such difference in atomic size and electronegativity may induce curvature in nanostructures of these materials. Electron localization function evidenced that the O–W bonds formed in the Janus structures combine covalent and ionic characters. Oxidation causes a band gap transition from direct-to-indirect in both Janus structures at different coverages, 1/9 ML O in WS_2_ and 1/3 ML O in WSe_2_, a fact that happens because the states at the Γ high symmetry point shift toward the Fermi energy, while the states at the K shift toward more negative energies. The capacity of oxidized Janus and nonoxidized pristine monolayers to trap ions present in seawater and brackish water was tested in the gas phase and under aqueous conditions. In the case of pristine monolayers, they interact strongly with Ca, K, Na, and Cl, while they depict lower adsorption energies for Mg ions. The data show that pristine monolayers are not selective to cations. In sharp contrast, the oxidized part of the Janus monolayers exhibits selective cation adsorption, forming chemical bonds with Ca, Mg, K, and Na. At the same time, Cl interacts with the surface through weak van der Waals forces. Such an effect is seen in the gas phase and also under aqueous conditions, where the ionic chemical bonds of cations with the surface were confirmed by using electron localization function analysis, and the weak Cl-surface interactions were evidenced using a noncovalent index analysis. Our results indicate that Janus TMDs can be used as efficient anodes for water remediation.

## Supplementary Material



## Notes

Electron localization function (Figures S1 and S2); effective band structures (Figures S3 and S4); projected densities of states (Figures S5 and S6); ion adsorption models (Figures S7–S10); and ion adsorption models under aqueous conditions (Figure S11) (PDF)
