# Supplementary material for: Atomistic Detail of the Formation of WSO and WSeO Janus Monolayers and Their Role for Cation Selection: Toward Effective Materials for Environmental Remediation
Source: ACS Omega. 2025 Sep 20;10(38):43580–90. doi: 10.1021/acsomega.5c03270 (PMC12489664; doi:10.1021/acsomega.5c03270)
Supplement: Supplementary file 1 [file ao5c03270_si_001.pdf]

# On the atomistic detail of the WSO and WSeO Janus monolayers formation and their role for cation selection: towards effective materials for environmental remediation

Jonathan Guerrero-Sanchez[1*], Dalia M. Muñoz-Pizza[2], Do Minh Hoat[3,4]

[1]Centro de Nanociencias y Nanotecnología, Universidad Nacional Autónoma de México, km.107, Apdo. Postal 14. Carretera Tijuana-Ensenada, Ensenada, Baja California, México

[2]Facultad de Ciencias, Universidad Autónoma de Baja California, Ensenada, Baja California, Mexico

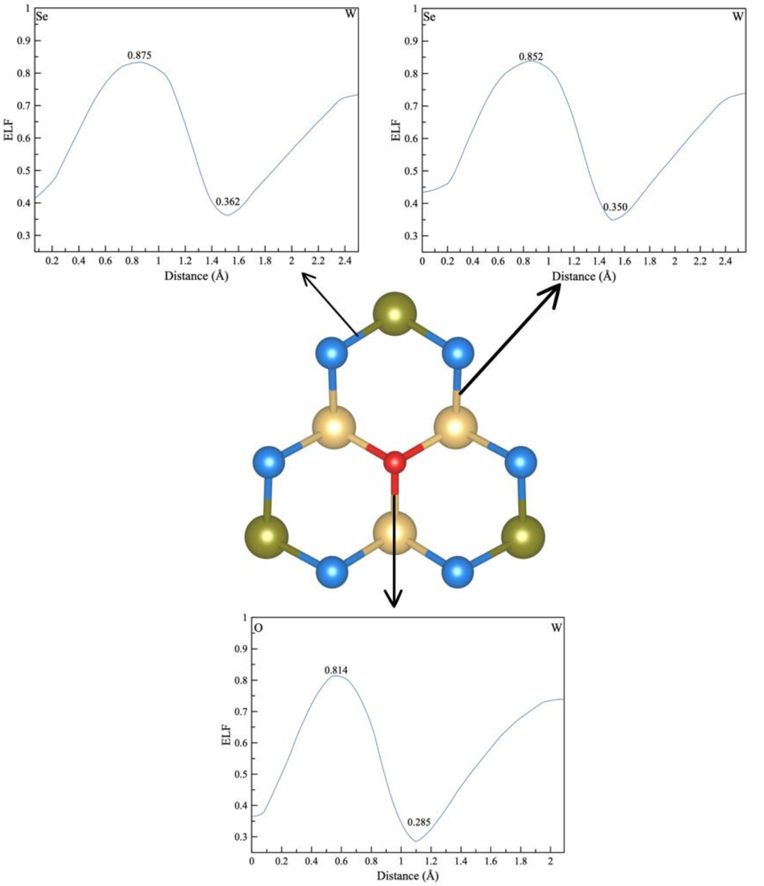

Figure S1. Electron Localization Function Line Profile for different bonds in the Oxygen incorporated into the $WS_2$ monolayer. Upper left part: line profile of the S-W bond far from the incorporated O atom. Upper right part: line profile of the S-W bond near the incorporated O atom, and the bottom center part depicts the line profile of the O-W bond. In all line profiles, the Ionic-Covalent character of the bonds can be observed.

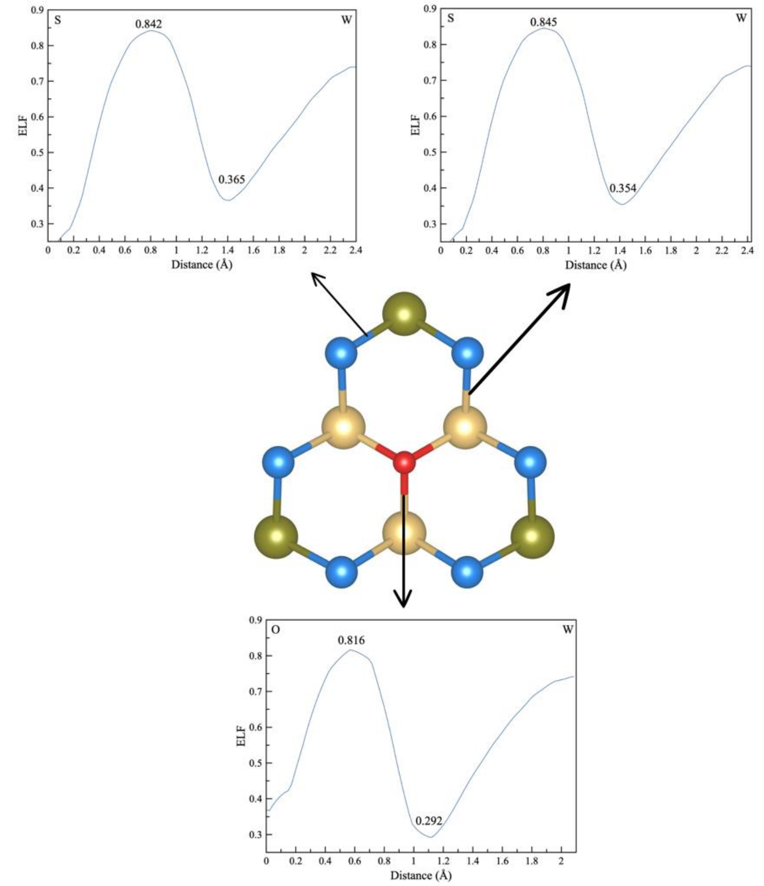


Figure S2. Electron Localization Function Line Profile for different bonds in the Oxygen incorporated into the $WSe_2$ monolayer. Upper left part: line profile of the Se-W bond far from the incorporated O atom. Upper right part: line profile of the Se-W bond near the incorporated O atom, and the bottom center part depicts the line profile of the O-W bond. In all line profiles, the Ionic-Covalent character of the bonds can be observed.

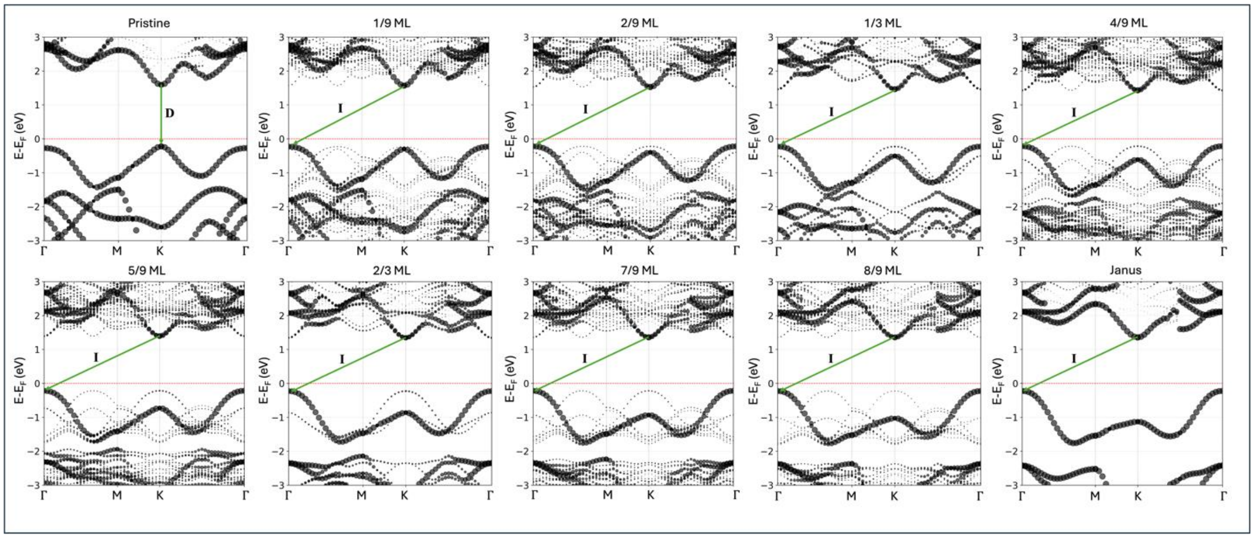


Figure S3. Effective band structures for different structures based on $WS_2$. From left to right, upper part: Pristine, 1/9 ML O, 2/9 ML O, 1/3 ML O, and 4/9 ML O. From left to right, lower part: 5/9 ML O, 2/3 ML O, 7/9 ML O, 8/9 ML O, and Janus WSO monolayer. The indirect-to-direct band gap transition occurs at 1/9 ML O coverage.

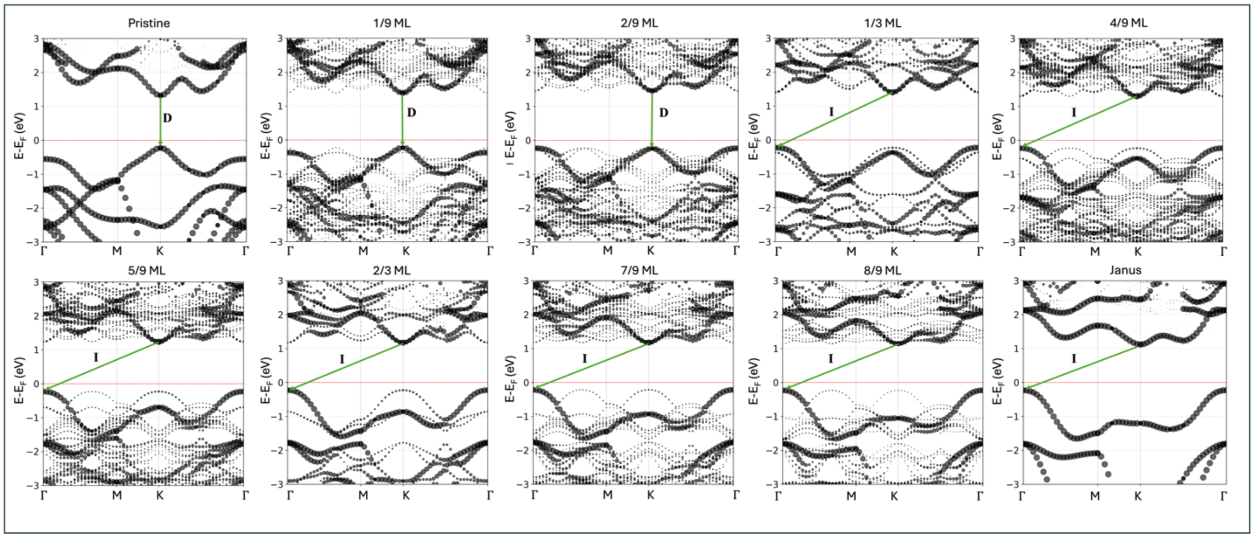


Figure S4. Effective band structures for different structures based on $WSe_2$. From left to right, upper part: Pristine, 1/9 ML O, 2/9 ML O, 1/3 ML O, and 4/9 ML O. From left to right, lower part: 5/9 ML O, 2/3 ML O, 7/9 ML O, 8/9 ML O, and Janus WSeO monolayer. The indirect-to-direct band gap transition happens at 1/3 ML O coverage. Before the transition, the band gap increases (1/9 ML O and 2/9 ML O)

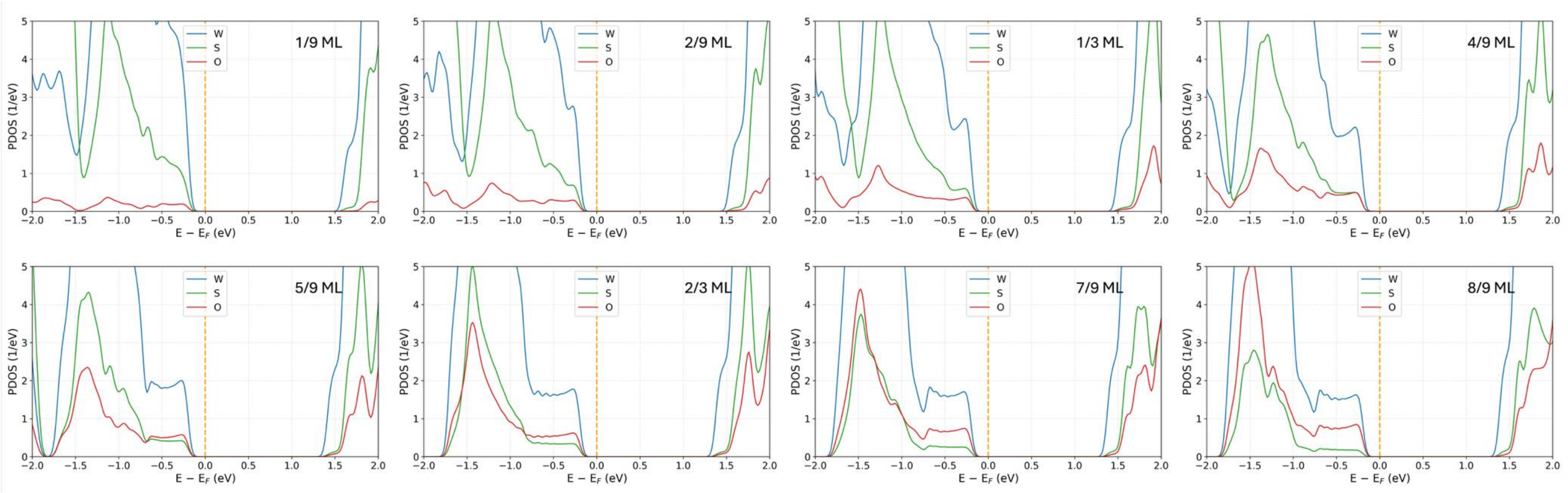


Figure S5. Upper part, from left to right 1/9, 2/9, 1/3, and 4/9 ML O incorporation, lower part, from left to right 5/9, 2/3, 7/9, and 8/9 ML O incorporation. Notice that from 1/9 ML O, a hybridization between O and W orbitals appears around -0.5 eV, and this hybridization remains and becomes stronger in the energy range between 0 and -0.5 eV.

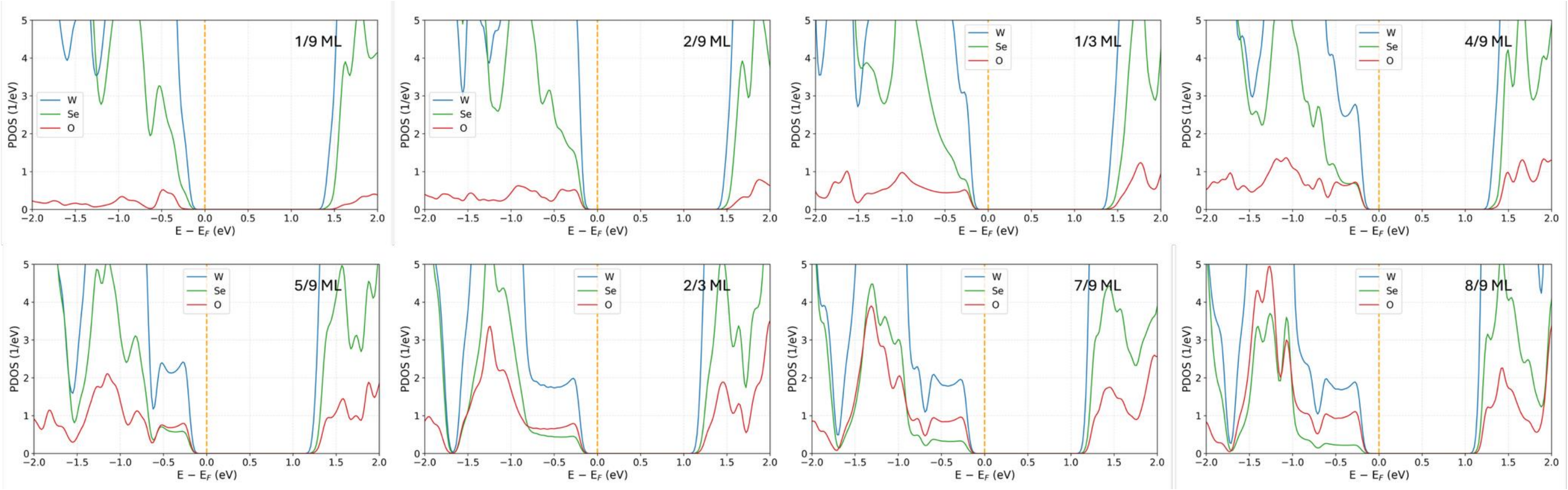


Figure S6. Upper part, from left to right 1/9, 2/9, 1/3, and 4/9 ML O incorporation into the $WSe_2$ monolayer, lower part, from left to right 5/9, 2/3, 7/9, and 8/9 ML O incorporation. Notice that from 1/3 ML O, a strong hybridization between O and W orbitals appears around -0.3 eV. This hybridization remains and becomes stronger in the energy range between 0 and -0.5 eV.

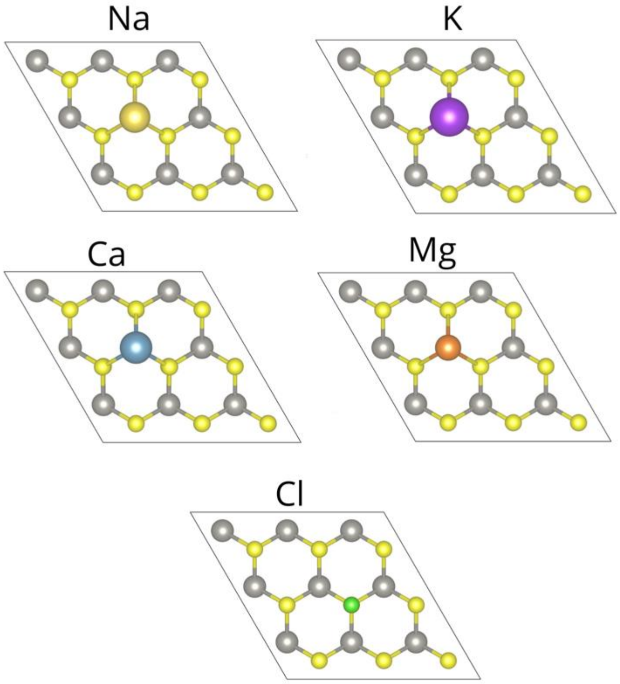


Figure S7. Adsorption models of different ions on $WS_2$ monolayer. Gray (W) and yellow (S).

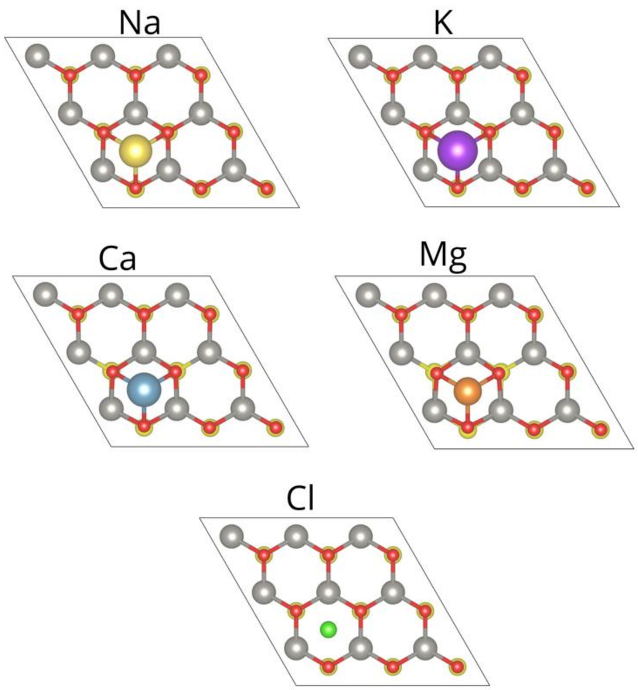


Figure S8. Adsorption models of different ions on WSO monolayer. Gray (W) and red (O).

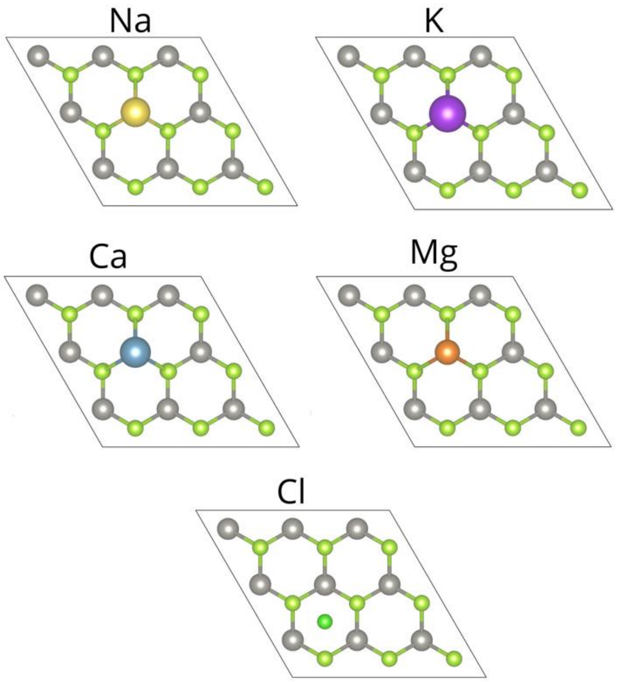


Figure S9. Adsorption models of different ions on $WSe_2$ monolayer. Gray (W) and light green (Se).

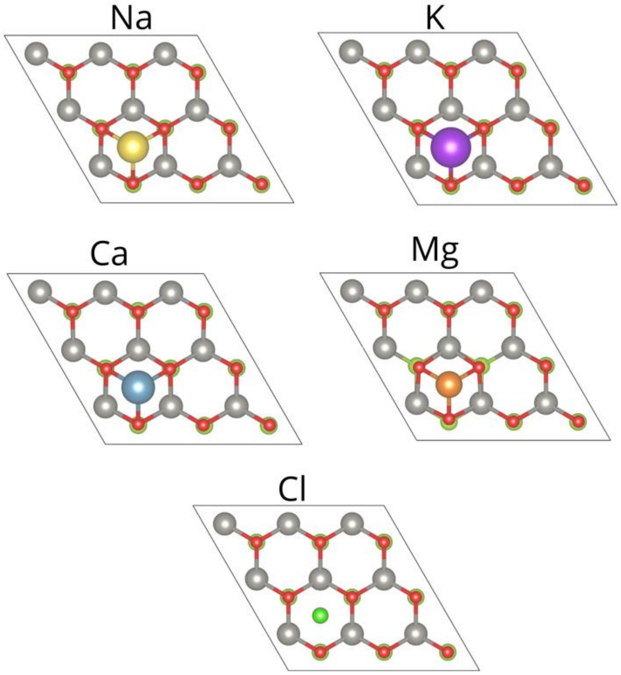


Figure S10. Adsorption models of different ions on WSeO monolayer. Gray (W) and red (O).

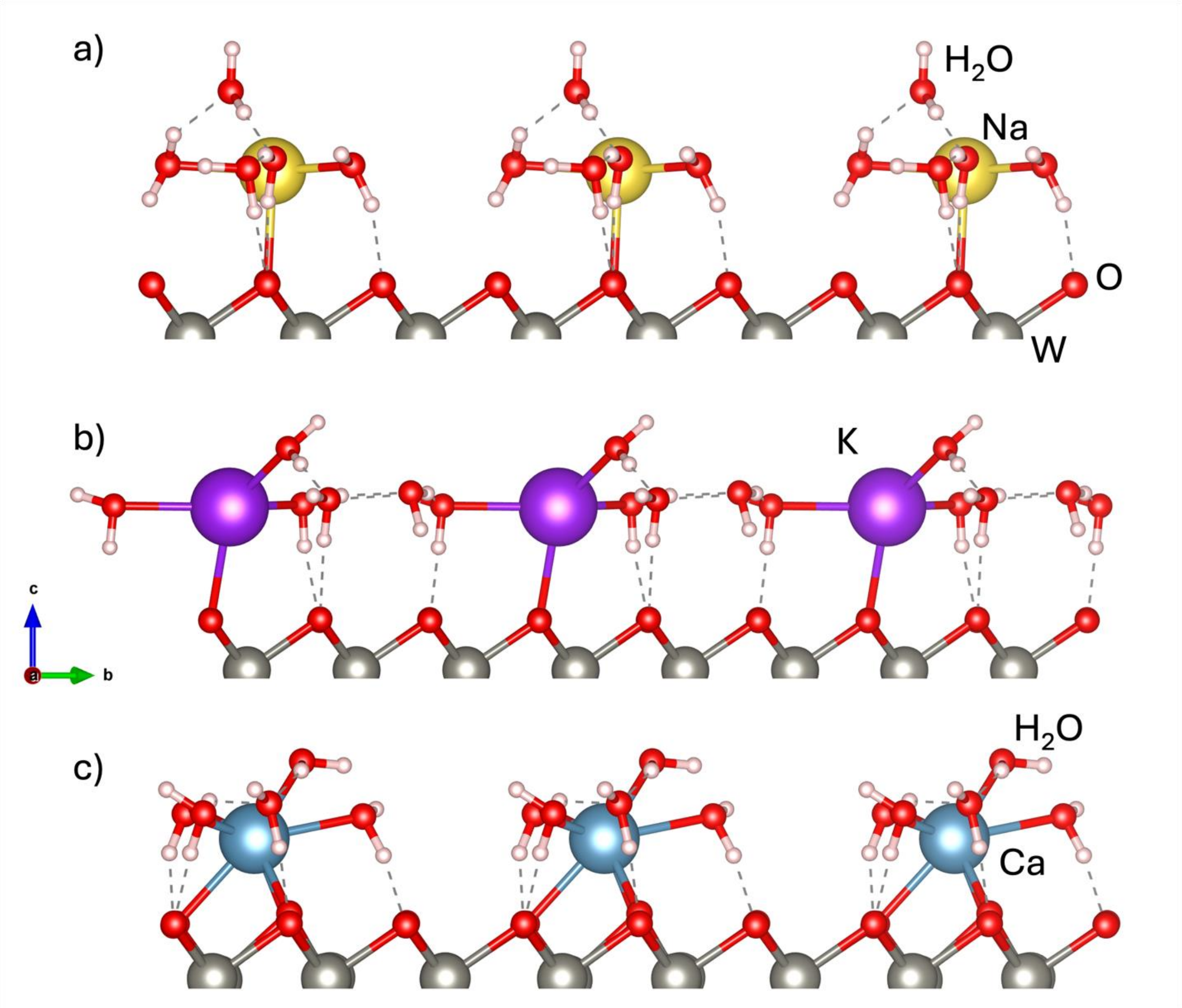


Figure S11. a) Na adsorption, b) K adsorption, and c) Ca adsorption under aqueous conditions. Gray spheres represent W atoms, yellow sulfur or selenium atoms, and the red ones are oxygen.

Figure S11 shows that adsorption under aqueous conditions also generates cationic species adsorbed to the surface, confirming the cation selection in the Janus monolayers. In the case of Na and K, the cation is forming single bonds with the O surface atoms. In the case of Ca, the cation is surrounded by four water molecules and is threefold bonded to the Janus monolayer. All systems were relaxed without imposing any constraints.
